# Books Reviewed

**Published:** 1862-07

**Authors:** 


### BOOKS REVIEWED.

*A Practical Guide on the Study of the Diseases of the Eye; their Medical and Surgical Treatment. By* HENRY W. WILLIAMS, M. D., *Fellow of the Massachusetts Medical Society, Honorary Fellow of the Rhode Island Medical Society, Member of the American Medical Association, etc., etc., etc. Boston:* TICKNOR & FIELDS. *New York:* SHELDON & CO. *Philadelphia:* J. B. LIPPINCOTT & CO., *and* LINDSAY & BLACKISTON, 1862.

Those acquainted with Dr. Williams, and who know the soundness of his judgment and practice, will not need any statement from us of the excellence of his book. They would be surprised were it otherwise.

Many of our readers will doubtless remember a small pamphlet published by him a few years since, containing an account of quite a large number of cases of iritis, from various causes, treated successfully without mercury. The results published were the more remarkable and important, inasmuch as iritis had always been pointed out as *the* disease above all others which clearly demonstrated the action of mercury in removing the effused products of inflammation; this action being not a matter of inference, but as was stated, being perceptible to the sight. Not only did the case in question illustrate the resolvent power of mercury, but it furnished an instance in which it was *essential.* One who reads attentively the pamphlet above named will, we think, entertain some doubt at least, of the certainty of this effect, as well as the necessity of this administration. The same disregard of previously formed opinious is a notable feature of the present volume. In proof of this we may mention the omission in a great degree of the long technical names of the various affections of the eye, which one finds in such abundance in the work of Dr. Jones—the comparatively light estimation which he puts upon nitrate of silver and acetate of lead, two remedies which have been largely relied upon and recommended heretofore—and his decided advocacy of simple treatment in many cases in which activity has been thought essential, as for instance in traumatic affections of the eye.

It appears to us to be one of the merits of the book that the various affections of the eye are clearly stated without unnecessary multiplication, or lengthened description, so that a practitioner can feel that the whole subject is, after all, in a narrower compass than we are led to think from the more elaborate works. As an instance, we may mention the chapter on cataract, in which we find fewer varieties, and indeed no mention whatever of lenticular cataract as a distinct variety.

The aid given by the ophthalmoscope in the examination of the eye, has added much to our knowledge of the actual changes which occur in its different tissues, and has undoubtedly somewhat simplified the catalogue of diseases, but still we feel inclined to give the author credit for something more than a judicious arrangement. We do not find in the description of the separate diseases of the eye, such numberless sub-divisions, that the mind is in some degree of confusion as to which division certain symptoms belong. In many works one may carefully study the subject of cataract, glaucoma and amaurosis, and after all fail to keep clearly before the mind the main distinctions between them. Amaurosis has been made to cover quite a broad field, including symptoms which can now be explained in some other manner—especially asthenopia, which we could hardly confound with it after a careful study of the book before us.

After a careful perusal we do not hesitate to declare this book of Dr. Williams, one of very great excellence. The simplicity and brevity of statement, which cannot fail to strike the attention, contribute so much to a clear and thorough comprehension of the subjects treated, while nothing is lightly passed over which is worthy of being noticed. While it is brief it is thorough. We have been much gratified with the chapter on "Affections of the cornea," a subject which is treated more concisely and clearly than we have ever before found it.

We may state as a proof of the able manner in which the author has performed his work, that one rises from its perusal with a feeling, that he has a clear notion of the range, and distinctions, of affections of the eye. And it may be safely assumed, that concise and clear statements, are sure evidence of clear and precise information, in the writer. Indeed the book clearly indicates an extensive and well-improved experience; the author evidently aiming to state his own convictions and observations rather than making a summary of what is known. We do not think that the probable result in all cases of diseases of the eye is elsewhere so well stated; and we are certain that no rules of treatment, at the same time so simple and precise, are laid down by any other author. Without, however, entering any further into the comparison of this, with other books, we will content ourselves with saying that in style, method and soundness of statement, it is entitled to be considered a superior book. We know of none which the reader may consult with equal advantage.

The appearance of the book is every way very elegant, and worthy of the excellent matter it contains. Such clear and distinct type, is of itself, almost enough to tempt one to read, irrespective of the instruction he may find in the contents of the volume.

---

*Hand-Book of Surgical Operations. By* Stephen Smith, M. D., *Surgeon to Bellevue Hospital. New York:* Bailliere Brothers, 440 *Broadway.*

Since the war has drawn many surgeons into active practice, separating them from their libraries and excluding the possibility of having in all cases the larger works upon surgery for ready reference, quite a number of smaller books have been published, to supply a want, which was early felt and expressed by the volunteer surgeons. The Sanitary Commission endeavored to supply this indication by publishing monographs upon some of the more common points of practice, and have succeeded in accomplishing what they proposed in remarkable degree.

Smith's Hand-book of Surgical Operations, has been prepared with reference to the wants of the medical staff of the Army, and is limited to those branches of operative surgery which are of most importance to military surgeons. The book is thus rendered convenient and portable, and may be carried as a reference and guide in all emergencies. Almost every important operation is represented by engravings which illustrate the subject to the fullest extent practicable, and add very much to the value of the work. Illustrations of instruments are also made, which represent the latest improvements, and constitute also an important addition. Though this hand-book of surgery is made to conform to the necessities of military surgery, still it is also a valuable hand-book of surgery in civil practice as well; and almost every operation in surgery is described with sufficient detail for the ordinary purposes of study and practice. As a hand-book of surgery, it stands at the head of a long list of similar books.

---

*A Manual of Medical Diagnosis, being an Analysis of the Signs and Symptoms of Disease. By* A. W. BARCLAY, M. D., *Fellow of the Royal College of Physicians; Assistant Physician to St. George Hospital. Second American, from the second and revised London edition. Philadelphia:* BLANCHARD & LEA, 1862.

Dr. Barclay has furnished a guide to the systematic study of disease which will be appreciated by every student of medicine; and will be found an invaluable aid to correct diagnosis. After speaking in general terms of the "Province of Diagnosis,—Methods," and,—plan of carrying on investigation, and giving the table of classification of diseases used in St. George's Hospital, our author proceeds to consider the "*General Condition of the Patient,*" speaking minutely of the value of what may be called the general symptoms, and the special indications which may be derived from them, singly, and in groups.

Chapters are devoted to febrile diseases—rheumatism and gout—diseases of adventitious origin—diseases of uncertain or variable seat—chronic blood ailments—depraved constitutional states—the quasi-nervous diseases—general examination of organs—semeiology of disease of the brain—diseases of the brain—diseases of the spinal cord—paralysis—neuralgia—examination of the chest—modifications of normal breath and voice sound, and of percussion resonance—superadded sounds, in their relation to altered breath and voice-sounds—diseases of respiratory organs—examination of the heart—diseases of the heart—diseases of the blood vessels—diseases of the mouth and pharynx—examination of the abdomen—diseases of the œsophagus and stomach—diseases of the intestinal canal—diseases of the peritoneum—diseases of the liver, spleen and pancreas—examination of the urine—diseases of the urinary organs—diseases of the ovaries—diseases of the uterus—diseases of the bones, joints and muscles—diseases of the skin and cellular tissue.

We have enumerated the various topics discussed in this book for the purpose of showing, somewhat, its scope and design. The divisions and subdivisions of these subjects are numerous, and embrace what is known upon the various points under consideration. It is written in fine, attractive style, and the attentive reader will not fail to be entertained while he is instructed. One of the chief excellencies of this book will be found to consist in the clear, distinct and truthful descriptions given of disease and the definite determination of the value of every symptom, tracing it carefully to its cause, and guarding against all sources of mistake as to its true import.

This book is a very valuable one to the profession; and while we most heartily commend it to our readers, regret exceedingly that we have not space to speak of it more at length, and give a better and more complete idea of its character and value.

---

